# Stability of class II subdivision malocclusion treatment with 3 and 4 premolar extractions

**DOI:** 10.1186/s40510-014-0067-4

**Published:** 2014-12-30

**Authors:** Guilherme Janson, Janine Araki, Sérgio Estelita, Leonardo T Camardella

**Affiliations:** Department of Orthodontics, Bauru Dental School, University of São Paulo, Alameda Octávio Pinheiro Brisolla 9-75, Bauru, São Paulo 17012-901 Brazil; Department of Orthodontics, Federal University of Rio Grande do Sul, Rua Ramiro Barcelos 2492, Porto Alegre, Rio Grande do Sul 90035-003 Brazil

**Keywords:** Asymmetry, Class II malocclusion, Extraction, Stability, Treatment

## Abstract

**Background:**

The purpose of this study was to compare the occlusal stability of class II subdivision malocclusion treatment with 3 and 4 first premolar extractions. A sample of 156 dental casts from 52 patients with class II subdivision malocclusion was divided into two groups according to the extraction protocol. Group 1 comprised 24 patients treated with 3 premolar extractions and group 2 included 28 patients treated with 4 premolar extractions.

**Methods:**

Peer assessment rating (PAR) indexes were measured on the dental casts obtained before (T1) and after treatment (T2) and at a mean of 6.9 years after the end of treatment (T3). The groups were matching regarding sex distribution, pretreatment, posttreatment and long-term posttreatment ages, and treatment and long-term posttreatment times. They were also comparable concerning the initial malocclusion severity and the occlusal results at the end of treatment. Stability evaluation was calculated by subtracting the posttreatment from the long-term posttreatment index values (T3 − T2). T tests were used to compare the amount and percentage of long-term posttreatment changes.

**Results:**

There were no intergroup differences regarding the amount and percentage of long-term posttreatment changes.

**Conclusion:**

Treatment of class II subdivision malocclusion with 3 and 4 premolar extractions have a similar long-term posttreatment occlusal stability.

## Background

Currently, most studies show that class II subdivision malocclusion is primarily caused by distal positioning of the mandibular first molar in relation to the maxillary first molar, on the class II side [1-7]. Secondarily, it can be consequent to mesial positioning of the maxillary first molar, in relation to the mandibular first molar, on the class II side [2]. As a result, most class II subdivision malocclusion patients present the mandibular dental midline displaced toward the class II side associated to the maxillary dental midline coincident to the midsagittal plane or with a mild deviation, which require asymmetric orthodontic approaches [1,2].

In patients with the mandibular first molar and dental midline displaced toward the class II side, the possible orthodontic treatment approaches include extractions of 3 or 4 premolars when some retraction of the profile is allowed [2,4,6-9]. The 3 premolar extraction (two maxillary premolars and one mandibular premolar on the class I side) protocol finishes with bilateral class I canine relationship, maintaining the original unilateral class II molar relationship on one side, and the 4 premolar extraction protocol (1 premolar per quadrant) requires finishing with bilateral class I canine and molar relationships. Without using skeletal anchorage devices, the 4 premolar extraction protocol requires more patient compliance in using class II and anterior diagonal intermaxillary elastics to obtain accurate occlusal outcome and coincidence of the maxillary and mandibular dental midlines [2,4,6,8,10,11].

In addition to a satisfactory occlusal outcome, long-term stability is one of the main treatment objectives. It has already been demonstrated that 3 premolar extractions has a greater occlusal success rate than 4 premolar extractions in the treatment of the above mentioned class II subdivision malocclusions [8,12]. However, long-term stability of cases finished with class II molar relationships is questionable [13,14]. Because the orthodontic literature is deficient in studies on the stability of class II subdivision treatment with 3 premolar extractions, the objective of this study is to compare the stability of patients with class II subdivision malocclusions treated with either 3 or 4 premolar extractions, in the long-term.

## Methods

Ethical approval was obtained from the Ethics Research Committee of Bauru Dental School, University of São Paulo, Brazil. Written informed consents were obtained from the patients for the publication of this report and any accompanying images.

**Figure 1:**
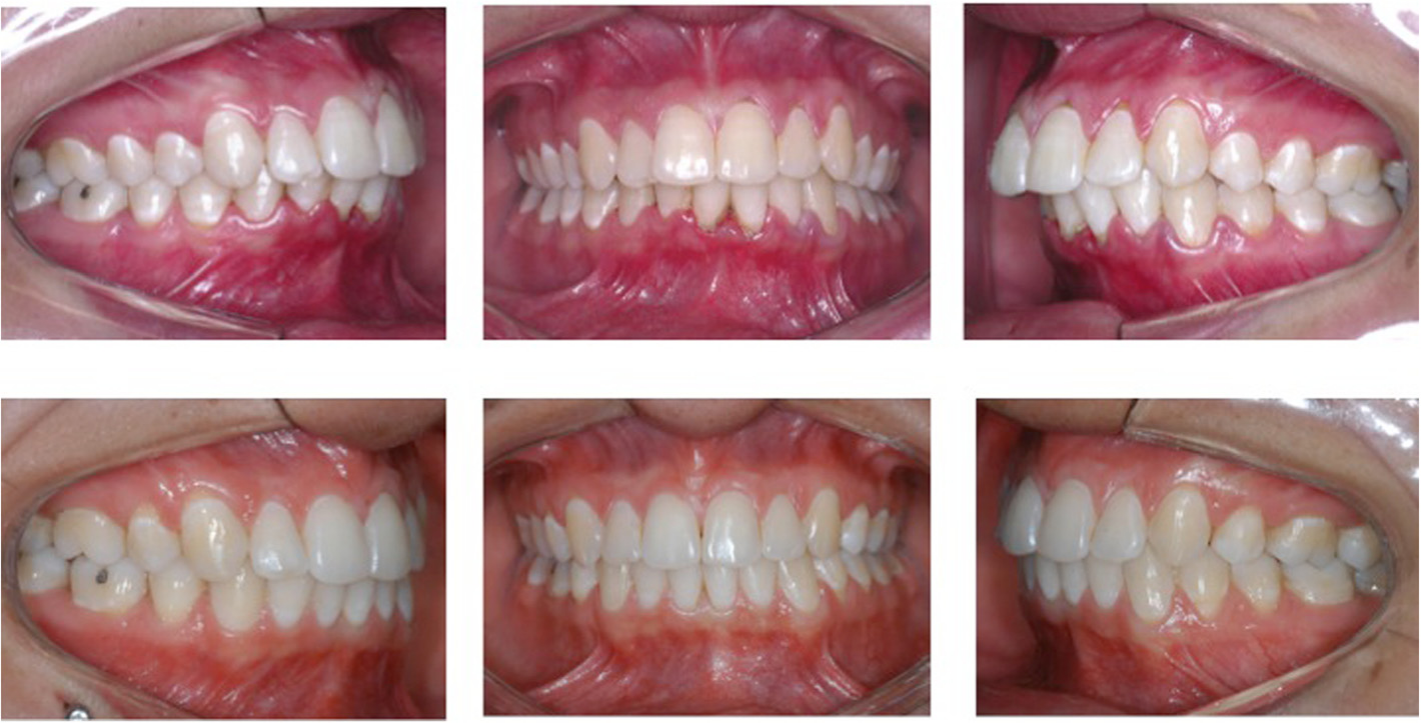
**Intraoral photographs of one patient treated with 3 premolar extractions.**

**Figure 2:**
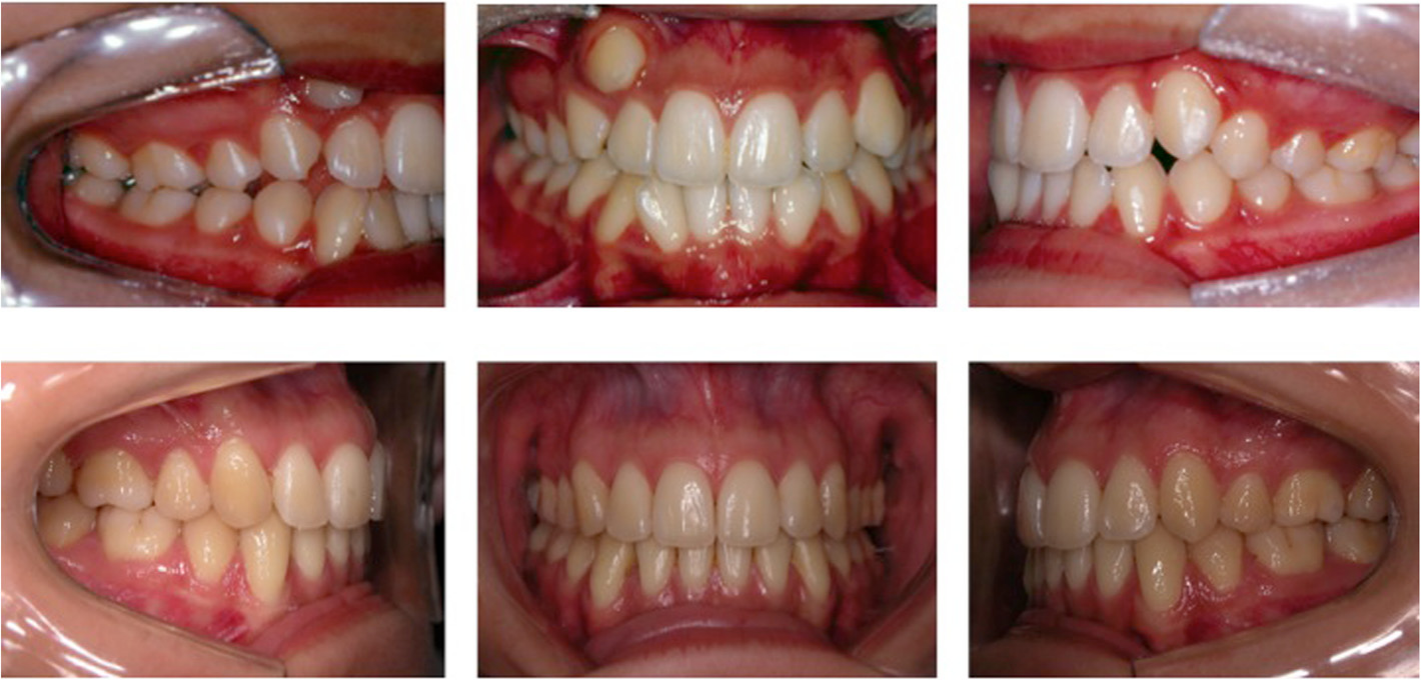
**Intraoral photographs of one patient treated with 4 premolar extractions.**

The sample was retrospectively selected from the files of the Orthodontic Department at Bauru Dental School, University of São Paulo, Brazil, which include over 4,000 documented treated patients. The pretreatment (T1), posttreatment (T2), and long-term posttreatment (T3 - at least after 2.13 years posttreatment) dental casts [15] of all patients who initially had class II division one subdivision malocclusion (complete class II molar relationship on one side and class I on the other side [16]) and were consecutively treated with 3 or 4 first premolar extractions and fixed appliances, were selected and divided into two groups (Figures 1 and 2). Additionally, all patients had all permanent teeth up to the first molars and no dental anomalies of number, size, and form and had no relevant facial asymmetry.

Sample size calculation was performed and showed that to detect a difference of 2.5 [17] in the peer assessment rating (PAR) index between two groups, with a standard deviation of 2.88 [18] at a significance level of 5% with a power of 80%, it was necessary to have a minimum of 23 subjects in each group.

Therefore, to increase the test power even more, group 1 consisted of 24 patients (9 male; 15 female) treated with 3 first premolar extractions (two maxillary premolars and one mandibular premolar on the class I side) at a pretreatment mean age (AGE1) of 13.54 ± 2.36 years (range, 9.50 to 21.06 years, Figure 1). The posttreatment age (AGE2) was 17.03 ± 2.65 years (range, 14.42 to 25.11 years) and the long-term posttreatment age (AGE3) was 23.45 ± 3.58 years (range, 18.33 to 29.87 years). The average treatment time (TT) was 3.48 ± 1.15 years (range, 1.67 to 5.56 years) and the average long-term posttreatment time (PT) was 6.43 ± 2.81 years (range, 2.13 to 10.98 years). Group 2 consisted of 28 patients (20 male; 8 female) treated with 4 first premolar extractions with a mean pretreatment age (AGE1) of 13.33 ± 1.34 years (range, 10.51 to 15.68 years), a mean posttreatment age (AGE2) of 16.31 ± 1.61 years (range, 14.01 to 20.68 years), and a long-term posttreatment age (AGE3) of 23.70 ± 4.17 years (range, 17.18 to 35.16 years, Figure 2). The average treatment time (TT) was 2.98 ± 1.24 years (range, 1.32 to 6.59 years) and the long-term posttreatment time (PT) was 7.40 ± 4.37 years (range, 2.47 to 20.42 years).

Orthodontic mechanics included fixed edgewise appliances, with 0.022 × 0.028-in conventional brackets and a usual wire sequence characterized by an initial 0.015-in Twist-Flex or 0.016-in Nitinol, followed by 0.016-, 0.018-, 0.020-, and 0.018 × 0.025- or 0.021 × 0.025-in stainless steel archwires (3 M Unitek, Monrovia, CA). Deepbites were corrected with accentuated and reversed Curve of Spee. In both groups, the anterior teeth were retracted en masse with a rectangular wire and elastic chains for overjet and unilateral class II canine correction. During retraction, an extraoral headgear, class II intermaxillary elastics or both were used to help maintain the class II molar relationship on one side in group 1 and to correct this anteroposterior relationship in group 2. No skeletal anchorage devices were used. A Hawley plate was used for retention during a mean period of 1 year in the maxillary arch, and a mandibular canine-to-canine fixed retainer was placed and recommended to be used for a mean period of 3 years.

### Dental casts and occlusal index

The PAR index [19] was calculated on the dental casts of each patient according to the American weightings suggested by De Guzman et al [20]. The index was ranked by scores for molar and premolar anteroposterior (AP) relationship, overjet (OJ), overbite (OB), crowding, and midline to quantify the initial malocclusion severity (PAR1), the treatment occlusal results (PAR2), the occlusal status at the long-term posttreatment stage (PAR3), the amount of treatment (PAR1-PAR2) and long-term posttreatment changes (PAR3-PAR2), and the percentage of PAR treatment and long-term posttreatment changes [20,21], which are better estimates of the occlusal changes [22]. Because the PAR index analyzes a set of occlusal characteristics at the same time and does not discriminate the participation degree of each in the total score, the scores obtained for each PAR component were also individually compared [18].

As previously mentioned, treatment changes were assessed using the numerical reduction in the index score (T1 to T2) and the percentage of reduction was determined by the formula T1 − T2/T1 × 100 [22,23]. Equally, the long-term posttreatment changes (stability evaluation) were measured using the numerical increase in the index score (T3 − T2) and the percentage of increase was determined by the formula T3 − T2/T1 × 100 [24]. The greater the numeric difference, the greater the treatment changes and the relapse. All measurements were performed with Mitutoyo calipers (Mitutoyo America, Aurora, IL), by one examiner (JA), where necessary.

### Error study

After a month interval, 42 pairs of dental casts were randomly remeasured by the same examiner (JA). Systematic errors were evaluated with dependent t tests at *p* < 0.05, and casual errors were calculated according to Dahlberg’s formula (Se^2^ = Σd^2^/2n), where Se^2^ is the error variance and d is the difference between two determinations of the same variable [25].

### Statistical analyses

Comparability of the groups concerning sex distribution was evaluated with chi-square tests. *T* tests were used to compare the group ages at T1, T2, and T3, the treatment and the long-term posttreatment times. This test was also used for intergroup comparison of the initial malocclusion severities (PAR1), the occlusal results (PAR2), and the occlusal status at the long-term posttreatment stage (PAR3) and to compare the PAR treatment and long-term posttreatment changes and the percentage of treatment and long-term posttreatment changes.

The occlusal results were obtained for each component of the PAR index at T2 and T3, and the posttreatment changes were individually compared between the groups with Mann-Whitney U tests. A nonparametric test was used because the values of each PAR component did not have normal distribution, according to Kolmogorov-Smirnov tests.

## Results

None of the variables presented statistically significant systematic errors, and the range of casual errors varied from 0.70 (PAR2) to 0.96 (PAR3).

The groups were comparable regarding sex distribution, pretreatment, posttreatment and long-term posttreatment ages, and treatment and long-term posttreatment times. They were also comparable concerning initial malocclusion severity (PAR1) and the occlusal results (PAR2). There were no intergroup differences regarding long-term posttreatment occlusal status (PAR3), PAR treatment and posttreatment occlusal changes, and the percentage of treatment and posttreatment occlusal changes (Tables 1 and 2).

**Table 1:** **Comparability of groups regarding sex distribution (chi-square test)**

**Group**	**Sex**		**Total (n)**
	**Male (n)**	**Female (n) (N)**	
Group 1 (3 premolar extractions)	9	15	24
Group 2 (4 premolar extractions)	20	8	28
Total	29	23	52

Chi-square = 0.47.

df = 1.

*p* = 0.49.

**Table 2:** **Intergroup comparison of the studied variables (t test)**

**Variable**	**Group 1 (3 premolar extractions) n = 24**		**Group 2 (4 premolar extractions) n = 28**		
	**Mean**	**SD**	**Mean**	**SD**	***p***
AGE1	13.54	2.36	13.33	1.34	0.68
AGE2	17.03	2.65	16.31	1.61	0.23
AGE3	23.45	3.58	23.70	4.17	0.82
Treatment time	3.48	1.15	2.98	1.24	0.14
Long-term posttreatment time	6.43	2.81	7.40	4.37	0.36
PAR1	20.46	8.64	18.11	7.88	0.31
PAR2	2.54	1.56	3.18	1.63	0.16
PAR3	4.04	2.12	3.86	2.10	0.75
PAR treatment changes	17.92	8.27	14.93	7.59	0.18
PAR long-term posttreatment changes	1.50	2.77	0.68	2.18	0.24
Percentage of PAR treatment changes (%)	86.04	8.46	80.94	10.66	0.07
Percentage of PAR long-term posttreatment changes (%)	9.37	16.90	4.98	16.08	0.34

AGE 1, PAR1: age and PAR index value at T1 - pretreatment; AGE 2, PAR2: age and PAR index value at T2 - posttreatment; AGE 3, PAR3: age and PAR index value at T3 - long-term posttreatment.

There were no intergroup differences regarding the several individual PAR components at the posttreatment and long-term posttreatment stages and during the long-term posttreatment period (Table 3).

**Table 3:** **Intergroup comparisons of the individual PAR components at T2 and T3 and during the long-term posttreatment period (Mann–Whitney U test)**

**Variable**	**Group 1 (3 premolar extractions) n = 24**	**Group 2 (4 premolar extractions) n = 28**	
	**Mean rank**		***p***
	**Mean (SD)**		
Posterior segments AP discrepancy at T2 (mm)	22.04	30.32	0.05
	1.08 (0.50)	1.43 (0.69)	
Posterior segments AP discrepancy at T3 (mm)	25.38	27.46	0.62
	1.33 (0.92)	1.39 (0.99)	
Posttreatment change in posterior segments AP discrepancy (T3 − T2) (mm)	28.25	25.00	0.44
	0.25 (1.15)	−0.04 (0.88)	
OJ at T2 (mm)	26.50	26.5	1.00
	0.00 (0.00)	00.00 (0.00)	
OJ at T3 (mm)	26.00	26.93	0.83
	0.00 (0.00)	0.04 (0.19)	
Posttreatment change in OJ (mm) (T3 − T2)	26.00	26.92	0.83
	−0.13 (0.34)	−0.21 (0.83)	
OB at T2 (mm)	26.63	26.39	0.96
	0.13 (0.34)	0.25 (0.80)	
OB at T3 (mm)	29.83	23.64	0.14
	0.42 (0.50)	0.18 (0.39)	
Posttreatment change in OB (T3 − T2) (mm)	29.38	24.04	0.21
	0.29 (0.55)	−0.07 (0.90)	
Crowding at T2 (mm)	26.50	26.50	1.00
	0.00 (0.00)	0.00 (0.00)	
Crowding at T3 (mm)	25.19	27.63	0.56
	0.13 (0.45)	0.25 (0.59)	
Posttreatment change in crowding (T3 − T2) (mm)	25.19	27.63	0.56
	0.13 (0.45)	0.25 (0.59)	
Dental midline at T2 (mm)	26.50	26.50	1.00
	0.00 (0.00)	0.00 (0.00)	
Dental midline at T3 (mm)	26.00	26.93	0.83
	0.00 (0.00)	0.04 (0.19)	
Posttreatment change in dental midline (T3 − T2) (mm)	26.00	26.93	0.83
	0.00 (0.00)	0.04 (0.19)	

AP, anteroposterior; OJ, overjet; OB, overbite; T2, posttreatment; T3, long-term posttreatment.

## Discussion

Considering the file with over 4,000 records of treated patients, from which the groups were selected, the 52 patients in this study might seem small. The reason for that were the rigid inclusion criteria applied since all patients should have a specific type of class II subdivision malocclusion (complete class II on one side and a class I on the other) at pretreatment and that they should have been treated with 3 or 4 premolar extractions. Moreover, the necessary records should be available, and the groups should be comparable regarding sex distribution, ages, and pretreatment and posttreatment occlusal characteristics (Tables 1 and 2).

Sample selection was based only on the initial anteroposterior dental relationship regardless of any other dentoalveolar or skeletal characteristic because we were only interested in evaluating the occlusal results and its stability. The anteroposterior dental relationship is more important than any other cephalometric parameter to quantify correction and stability of the occlusal anteroposterior discrepancy which was the main objective of the study [8,21]. Cephalometric dentoalveolar or skeletal characteristics are important when evaluating esthetic results, which were not the focus of this investigation [26]. Besides, the cephalometric characteristics have no influence in the occlusal results [27,28].

As the occlusal statuses at the long-term posttreatment stage, the PAR long-term posttreatment changes, and the percentage of PAR long-term posttreatment changes were similar; it can be concluded that treatment of class II subdivision malocclusion with 3 and 4 premolar extractions have a similar long-term posttreatment stability (Table 2). As previously mentioned, these results could not be compared with others because the literature is deficient in investigations on the stability of class II subdivision malocclusions treated with 3 premolar extractions. These results support previous investigations which demonstrated similar stability in complete class II malocclusions treated with 2 and 4 premolar extractions and 2 maxillary premolar extractions and non-extraction protocols [24,29,30]. Therefore, these studies do not confirm the idea that a posttreatment class II molar relationship has questionable long-term stability [13,14]. Class II malocclusion correction stability is not related to the treatment protocol or to the extracted teeth [24,29,30].

The occlusal statuses at the long-term posttreatment stage showed reasonable occlusion in both groups (Table 2). Occlusions with PAR indexes smaller than 5 are considered satisfactory [19]. In this study, the mean percentage of long-term posttreatment changes ranged from 4.98 to 9.37 in 6.9 years (Table 2), which is satisfactory as compared to other study that observed 33% of relapse, in 10 years, in general orthodontic treatment [31].

All PAR components were similar between the groups at the posttreatment and long-term posttreatment stages as well as during the long-term posttreatment period (Table 3). This confirms that a class II molar relationship that was kept unchanged during treatment remains stable, which agrees with other stability investigations [24,29,30].

In the long-term posttreatment stage (T3), some patients in both groups were still presenting bonded canine-to-canine retainers (18 patients in each group); however, the presence of a canine-to-canine fixed mandibular retainer does not impair this comparison because the PAR index evaluates the overall occlusion and not only the mandibular anterior crowding. Besides, mandibular crowding does not constitute a PAR component [19,20].

This study showed that long-term stability of class II subdivision malocclusion is similar when treated with 3 or 4 premolar extractions. Considering the greater occlusal success rate and less patient compliance necessary in the 3 premolar extraction protocol [8], this should be the preferred treatment option in class II subdivision malocclusions in which the mandibular midline is deviated in relation to the sagittal midplane, in facial profiles that accept extractions.

## Conclusion

Occlusal long-term stability of class II subdivision malocclusion treatment with 3 or 4 premolar extractions is similar.
